# Supplementary figures and images for: NRAS is unique among RAS proteins in requiring ICMT for trafficking to the plasma membrane
Source: Life Sci Alliance. 2021 Feb 12;4(5):e202000972. doi: 10.26508/lsa.202000972 (PMC7893820; doi:10.26508/lsa.202000972)

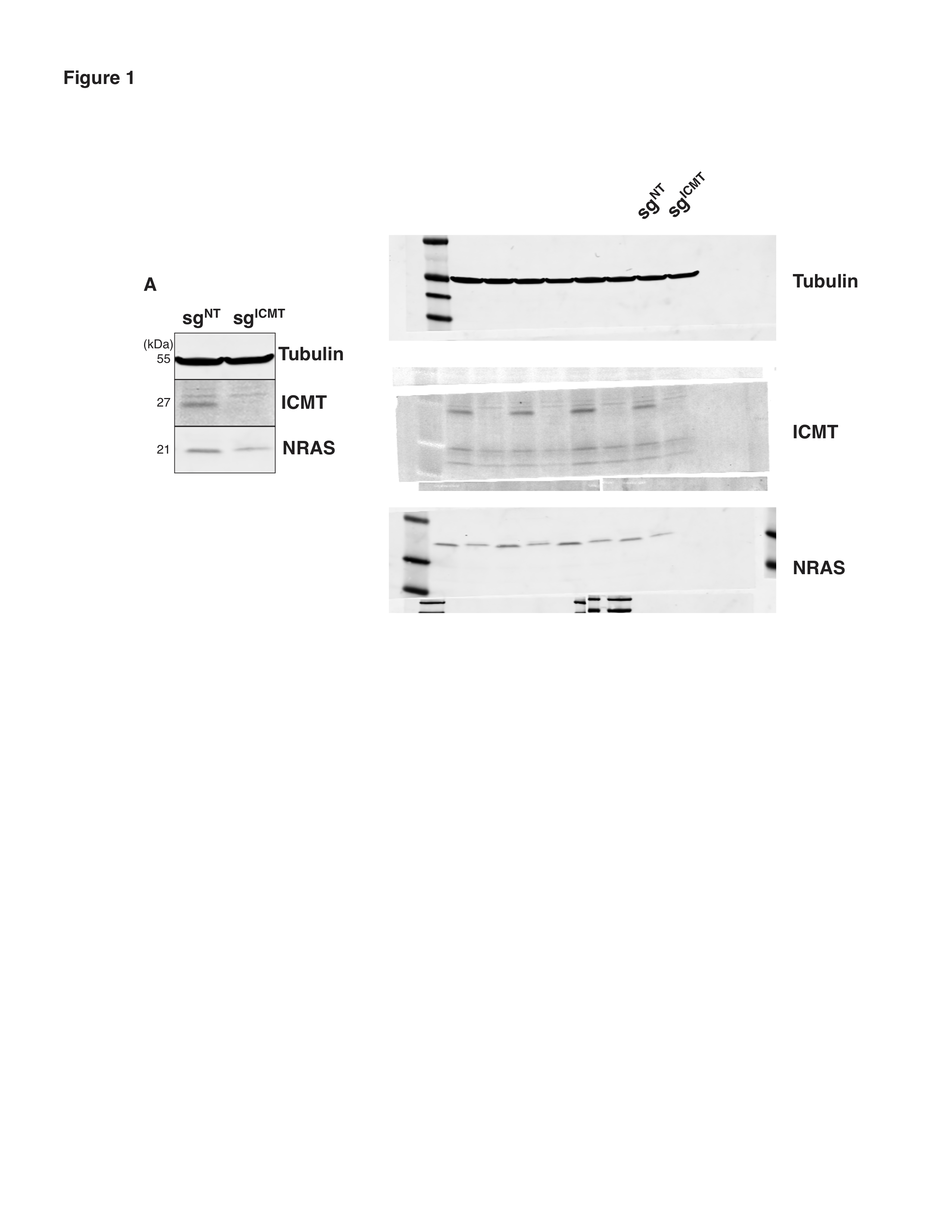

Supplement: Supplementary file 1 [file LSA-2020-00972_SdataF1.1.tif]

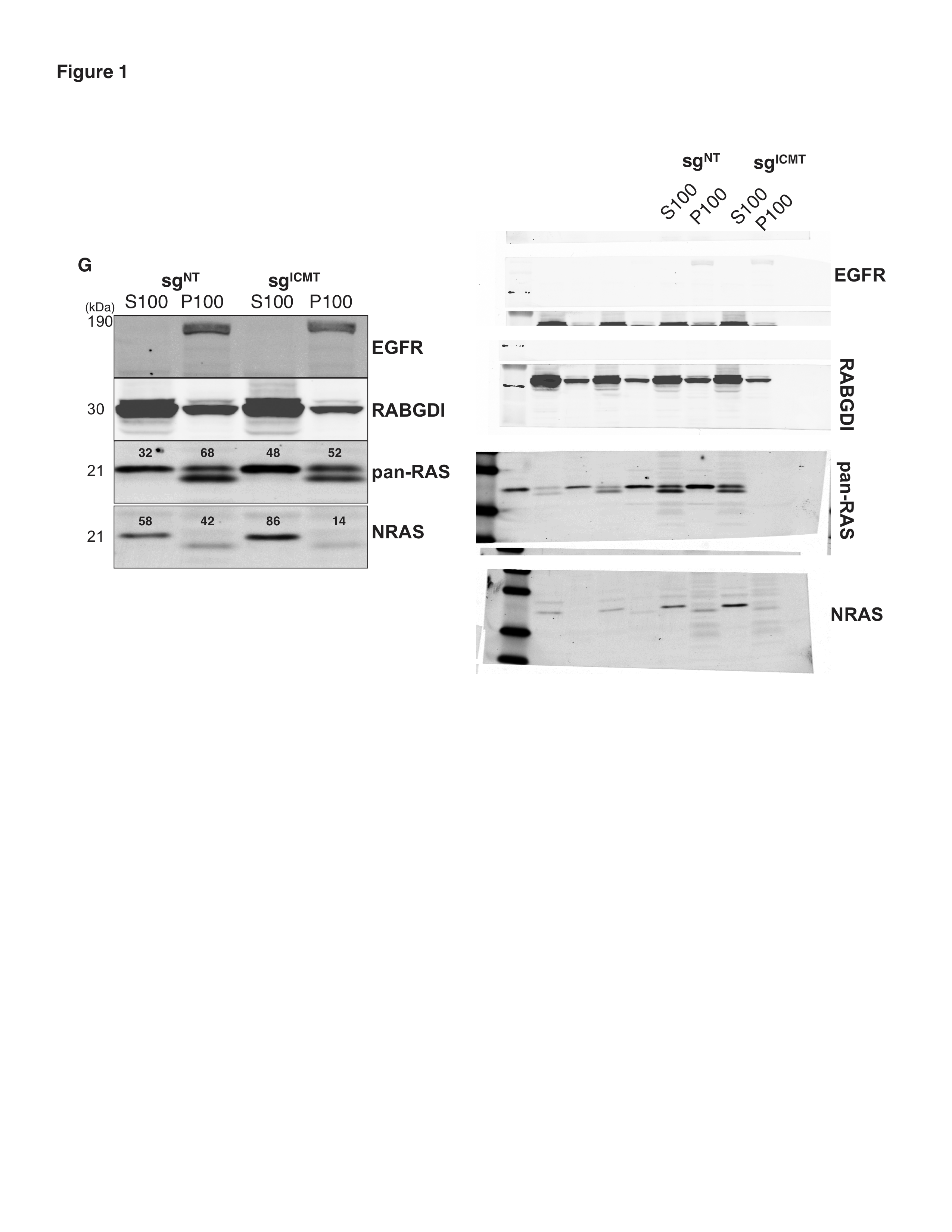

Supplement: Supplementary file 2 [file LSA-2020-00972_SdataF1.2.tif]

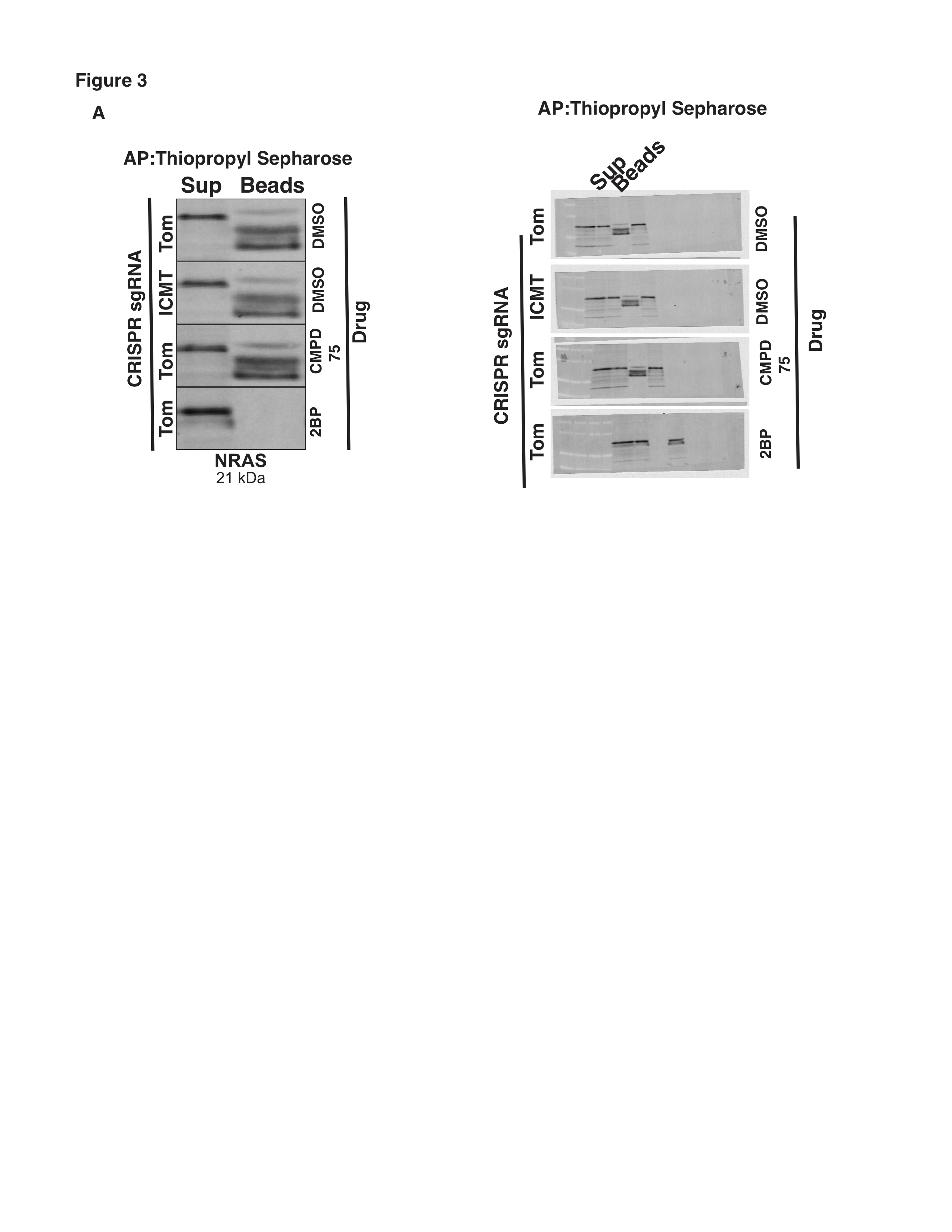

Supplement: Supplementary file 3 [file LSA-2020-00972_SdataF3.1.tif]

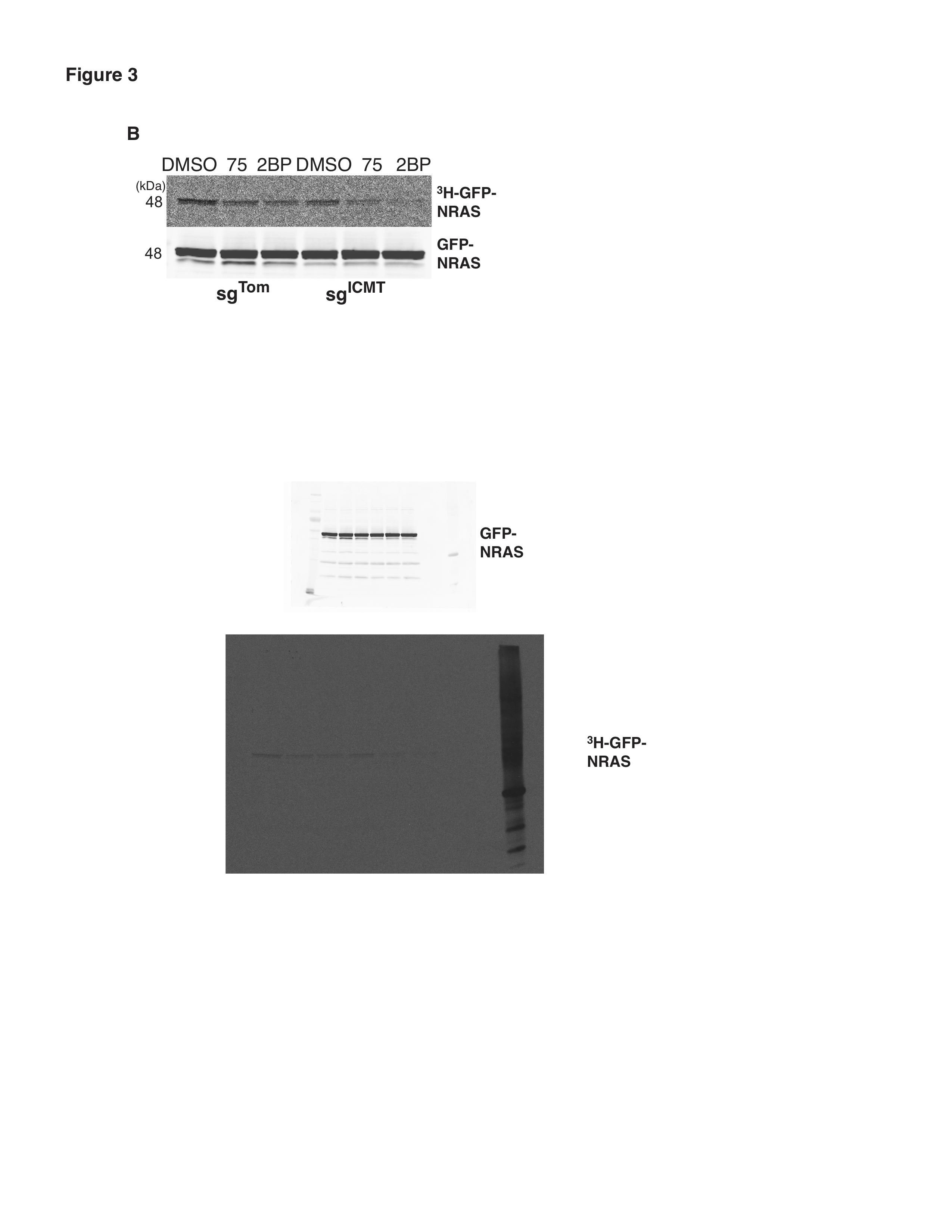

Supplement: Supplementary file 4 [file LSA-2020-00972_SdataF3.2.tif]
